# Migratory biliary stent resulting in colonic perforation: a rare complication and review of literature

**DOI:** 10.1093/jscr/rjad109

**Published:** 2023-03-18

**Authors:** Zachary Wilson, Jason Diab, Soni Putnis

**Affiliations:** Wollongong Hospital, NSW, Australia; Wollongong Hospital, NSW, Australia; School of Medicine, University of New South Wales, Sydney, Australia; School of Medicine, University of Notre Dame, Sydney, Australia; Wollongong Hospital, NSW, Australia

## Abstract

Biliary stent insertion during endoscopic retrograde cholangiopancreatography is used as a therapeutic intervention allowing flow of bile into the duodenum. In rare circumstances, distal gastrointestinal perforation can be attributed to a migrated biliary stent, with the most common site being the sigmoid colon. In these cases, surgical and/or endoscopic intervention may be required. We report a case of a 98-year-old male presenting with small bowel obstruction secondary to migrated plastic and metal biliary stents placed for acute biliary pancreatitis. Due to advanced age and high-risk multiple comorbidities, conservative management was undertaken. The patient was discharged after 5 days after ongoing pain and obstipation with palliative care services in place.

## INTRODUCTION

Biliary stents are used in the management of obstructive jaundice for both benign and malignant pathological processes [1]. There are many biliary stents available differing by size, design and material (plastic, polyethylene, Teflon or metal) [2]. Common complications include stent occlusion, cholangitis, bleeding and pancreatitis. Less common complications for biliary stents include dislocation and migration of biliary stents estimated to occur in 5–10% of patients [3]. Displaced stents may migrate distally to the colon; however, most will spontaneously pass without any complication [4]. In less common circumstances, distal bowel perforation is a rare complication of migrated biliary stents and may warrant surgical or endoscopic intervention [5]. We present a case of a migrated metal and plastic biliary stent causing small bowel obstruction leading to bowel perforation.

## CASE REPORT

**Figure 1:**
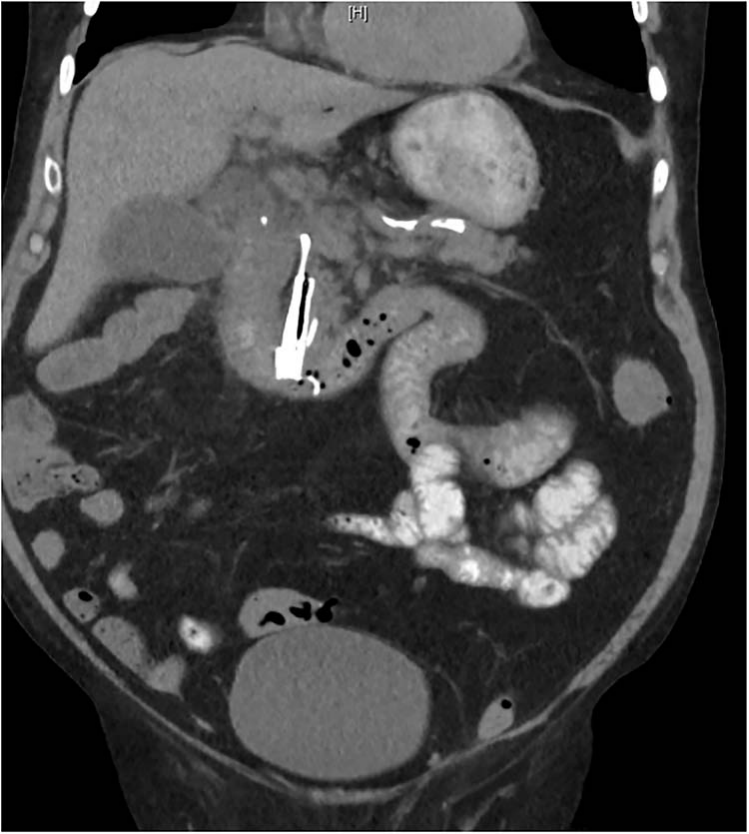
Abdominal CT showing a metallic biliary stent in distal CBD.

A 98-year-old male from a high-level care nursing home presented to the emergency department with abdominal pain, vomiting and faecal incontinence. His medical history included atrial fibrillation, transient ischaemic attack, chronic kidney disease, peripheral vascular disease, hypertension, gout and osteoarthritis. His surgical history included previous endoscopic retrograde cholangiopancreatography (ERCP) for acute biliary pancreatitis 30 months prior with insertion of a 4 cm × 10 mm metal biliary stent with an internal anchoring 7 Fr × 4 cm double pigtail plastic stent (Fig. 1). His Charlson comorbidity index was 3 with an American Society of Anaesthesiologists’ (ASA) classification of IV.

**Figure 2:**
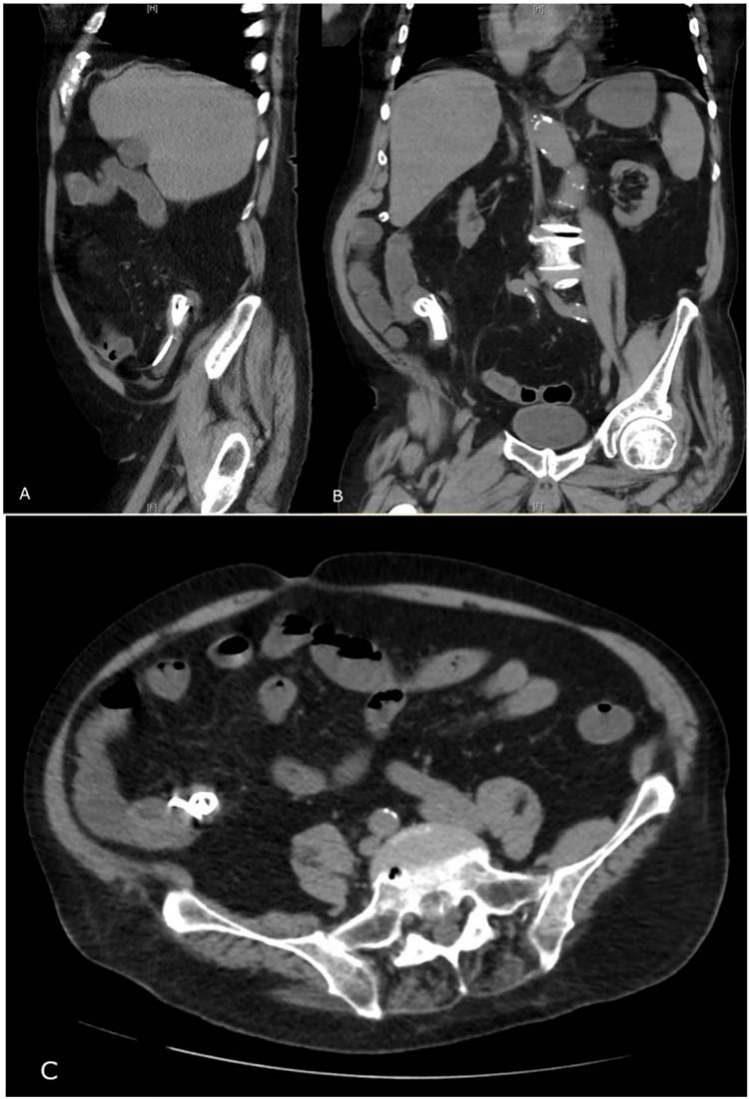
Abdominal CT showing migrated biliary stent in sagittal (**A**), coronal (**B**) and transverse (**C**) views.

On presentation, there was no signs of shock, with a blood pressure of 152/70, heart rate of 88 and oxygen saturations of 94% on room air. A temperature of 38.1°C, however, was noted and physical examination revealing a distended abdomen with right lower quadrant tenderness. He had no peritonism and digital rectal examination showed an empty rectum. Biochemical investigations showed a white blood cell count of 20.00 × 10^9^/L, C-reactive protein level of 126 mg/L, eGFR of 32 ml/min/1.73 m^2^, creatinine of 153 umol/L; he had normal liver function tests, coagulation profile and electrolytes. A computed tomography (CT) abdomen and pelvis with intravenous contrast revealed a distally migrated metallic biliary stent located in the terminal ileum with mildly dilated loops of distal small bowel indicating early or incomplete small bowel obstruction (Fig. 2).

**Figure 3:**
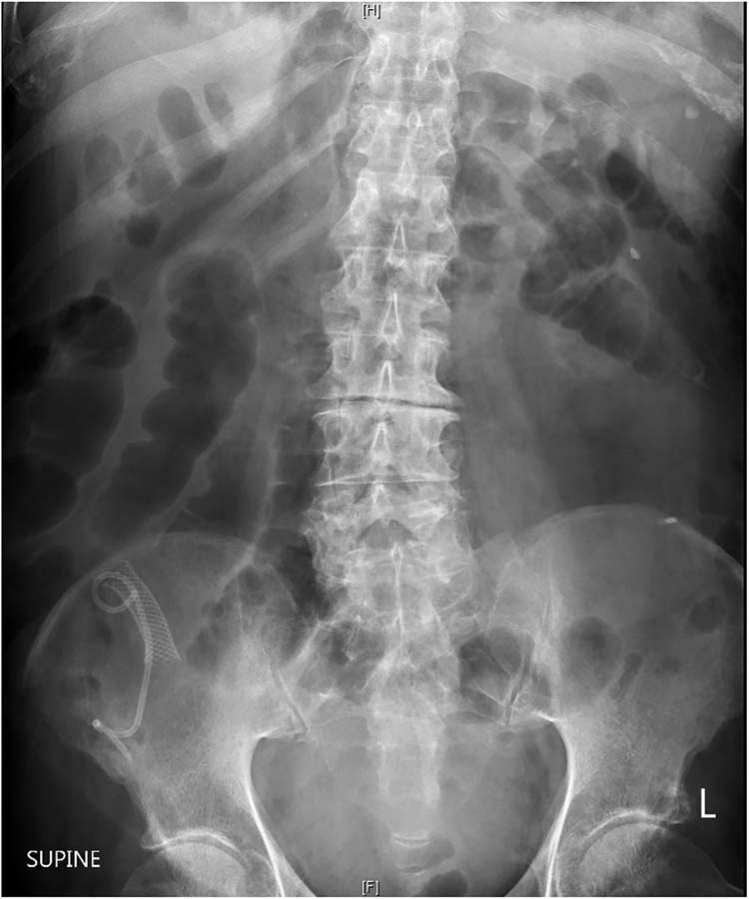
Abdominal radiography showing plastic and metallic biliary stents projected over the right iliac bone and dilated loops of small bowl throughout the abdomen and pelvis.

After a multidisciplinary consultation involving medical, geriatrics and surgical teams, the patient and family collectively decided for conservative management given age and high-risk multiple comorbidities. He was managed with intravenous fluids, broad spectrum Gram positive and Gram negative intravenous antibiotics, nasogastric tube and an indwelling catheter for 48 h. He progressed with minimal pain over the next 24 h and passed flatus. Subsequent plain abdominal radiography 2 days after presentation confirmed the presence of migrated metal and plastic biliary stents in the terminal ileum with dilated loops of small bowel suggestive of ongoing bowel obstruction (Fig. 3). After 5 days of not opening bowels and ongoing pain, he was transferred back to a nursing home facility with palliative care services in place. The patient passed away 3 weeks after transferring back to the nursing home facility with a suspected bowel perforation and no documentation of the stents having been passed.

## DISCUSSION

ERCP has evolved as a previously diagnostic to predominantly therapeutic intervention in the management of pancreaticobiliary disorders. It is most commonly utilized for the removal of common bile duct (CBD) stones and relief of obstructive jaundice [7]. As an advanced endoscopic procedure, it allows gastroenterologists and specialized surgeons to pass instruments into the biliary and pancreatic ducts permitting radiographic visualization and permitting flow into the duodenum [8]. A wide range of plastic and metal stents of differing designs are available for a board range of biliary and pancreatic conditions [9]. In a meta-analysis, metal stents in cases of malignant biliary obstruction led to longer stent patency and symptom-free survival at 6 months [6]. The use of plastic stents is common due to easy insertion/removal and lower costs, but have a higher occlusion rate which may lead to complications such as recurrent jaundice, pruritus and cholangitis [10]. The European Society for Gastrointestinal Endoscopy recommends that for benign biliary strictures and removal of CBD stones, placement of temporary plastic biliary stents is recommended with metal stents as an alternative [11].

The most frequent complications associated with stent insertions are occlusion with subsequent cholangitis or tumour over growth in cases of malignant lesions. Migrated biliary stents carry complications including fistula and/or abscess formation, obstruction and perforation of the gastro-intestinal tract in <1% of cases [2, 12]. In a multi-centre retrospective study, Emara et al. concluded that there is increased risk of stent migration associated with dilated CBDs, longer biliary stents and distal benign biliary strictures. Along with strictures that are dilated prior to stent insertion or if sphincterotomies have been performed during the procedure [13]. In cases of a benign biliary strictures, the aforementioned study recommended use of a maximum number of plastic stents may reduce the risk of migration when compared with insertion of a single plastic stent [14].

Perforation of the colon is a rare complication associated with a migrated biliary stent, which may warrant surgical and/or endoscopic intervention. The literature reports the sigmoid colon as the most common large bowel segment involved (Table 1). Endoscopic options for stent retrieval may be indicated in intraluminal cases and aid in facilitating shorter hospital stays. Surgical intervention is indicated in cases of perforations causing peritonitis, abscess or fistulas [1, 6]. The most common reported risk factor includes diverticulosis, followed by adhesions, hernia or strictures. These have been shown to increase the risk of perforation from a migrated biliary stent and patients with such factors should be counselled accordingly [5].

**Table 1:** A literature review of case reports on colon perforation due to migrated biliary stent

Author	Year	Age	Sex	Stent	Material	Indication	Time to migration	Risk factors	Presenting complaint	Perforation location	Management	Length of stay
D’Costa [16]	1994	73	M	10 Fr 10.5 cm	Plastic	CBD malignancy	Unknown	N/A	Abdominal pain	Ascending colon	Surgical	Unknown
Baty [17]	1996	86	F	Unknown	Polyethylene	Pancreas head cancer with CBD invasion	1 month	Diverticulosis	Abdominal pain	Sigmoid diverticula perforation	Sigmoidectomy	10 days
Schaafsma [18]	1996	77	F	Straight	Unknown	Acute Cholangitis with CBD stone	6 months	Diverticulosis	Abdominal pain	Sigmoid diverticula perforation	Surgical	Unknown
Lenzo [19]	1998	82	F	10 Fr 7.5 cm straight	Polyethylene	Acute Cholangitis with CBD stone	1 month	Diverticulosis	Abdominal pain	Sigmoid diverticula perforation	Surgical primary closure	11 days
Størkson [20]	2000	86	M	7 Fr 5 cm straight	Plastic	Acute Cholangitis with CBD stone	2 years	Unknown	Unknown	Sigmoid	Surgical primary closure	Unknown
Figueiras [21]	2001	47	M	10 Fr 10 cm straight	Polyethylene	Cholangitis – Benign stricture	3 months	N/A	Abdominal pain, PR bleeding	Splenic flexure	Removal through colocutaneous fistula	Unknown
Klein [22]	2001	70	F	7 Fr 5 cm straight	Teflon	CBD stone	3 years	Diverticulosis	Abdominal pain	Sigmoid diverticula perforation	Surgical	10 days
Elliot [23]	2003	80	F	10 Fr 10 cm straight	Unknown	Acute Cholangitis with CBD stone	4 months	N/A	Abdominal pain	Sigmoid	Hartmann procedure	Unknown
Diller [24]	2003	58	M	7 Fr 10 cm straight	Teflon	Post Liver Transplant bile duct stricture	1 month	Diverticulosis	Unknown	Sigmoid diverticula perforation	Sigmoidectomy	Unknown
Wilhelm [4]	2003	85	F	Straight	Unknown	CBD stone	18 months	Diverticulosis	Pneumaturia	Sigmoid diverticula perforation with colovesicular fistula	Sigmoidectomy	10 days
Anderson [25]	2007	80	F	Straight	Unknown	CBD stone	5 months	Diverticulosis	Leg pain and hip stiffness	Sigmoid diverticula perforation	Endoscopic removal	Unknown
Namdar [2]	2007	65	F	12 Fr 10 cm straight	Plastic	Post cholecystectomy bile leakage	3 months	N/A	Abdominal pain	Rectum	Rectal resection	7 days
Bagul [15]	2010	79	F	10 Fr 9 cm straight	Plastic	Post cholecystectomy bile duct stricture	3 months	Diverticulosis	Left groin swelling	Sigmoid diverticula perforation	Endoscopic removal	Unknown
Jafferbhoy [12]	2011	82	F	7 Fr 7 cm straight	Plastic	Post cholecystectomy bile leakage	3 months	Diverticulosis	Left iliac fossa pain	Sigmoid diverticula perforation	Endoscopic removal and clip closure	2 days
Lankisch [26]	2011	65	F	10 Fr 10 cm straight	Plastic	Pancreas head cancer with CBD invasion	2 weeks	N/A	Abdominal pain	Sigmoid	Surgery	Unknown
Malgras [27]	2011	73	Unknown	10 Fr 5 cm straight	Plastic	Pancreas head cancer with CBD invasion	15 days	Diverticulosis	Unknown	Sigmoid diverticula perforation	Hartmann procedure	Unknown
Wagemakers [28]	2011	76	F	Unknown	Plastic	CBD stone	1 month	Diverticulosis	Pneumaturia, fecaluria, persistent UTI	Sigmoid diverticula perforation	Sigmoidectomy	8 days
Alcaide [29]	2012	73	M	10 Fr 12 cm straight	Plastic	CBD stone with benign biliary stricture	15 days	Diverticulosis	Left iliac fossa pain	Sigmoid diverticula perforation	Endoscopic removal and clip closure	14 days
Kittappa [30]	2013	58	F	Unknown	Plastic	Post cholecystectomy bile leakage	18 months	Diverticulosis	Abdominal pain	Sigmoid diverticula perforation	Hartmann procedure	Unknown
Jones [31]	2013	66	M	Straight	Plastic	Post cholecystectomy bile duct stricture	3 months	N/A	Right upper quadrant pain	Caecum	Endoscopic removal	1 day
Konstantinidis [32]	2014	69	F	Straight	Plastic	CBD stone	2 months	N/A	Abdominal pain	Sigmoid	Surgical primary closure	7 days
Mady [33]	2015	Unknown	M	Unknown	Plastic	Pancreas head cancer with CBD invasion	1 month	Diverticulosis	Peritonitis and Shock	Sigmoid diverticula perforation with pelvic abscess	Hartmann procedure	Unknown
Virgilio [34]	2015	Unknown	F (Case 1)	Unknown	Plastic	CBD stone	Unknown	Diverticulosis	Abdominal pain	Sigmoid diverticula perforation	Hartmann procedure	Unknown
		Unknown	F (Case 2)	12 Fr 12 cm straight	Plastic	CBD stone	Unknown	Diverticulosis	Abdominal pain	Sigmoid diverticula perforation	Endoscopic removal	Unknown
Chittleborough [35]	2016	73	M	10 Fr 5 cm straight	Plastic	Acute Cholangitis with CBD stone	3 months	Diverticulosis	Abdominal pain	Sigmoid diverticula perforation	Hartmann procedure	18 days
Chou [36]	2017	85	F	Unknown	Plastic	CBD stone	Unknown	N/A	Asymptomatic	Sigmoid	Endoscopic removal and clip closure	2
Siaperas [37]	2017	75	F	Straight	Plastic	Post cholecystectomy bile duct stricture	1 month	Diverticulosis	Abdominal pain	Sigmoid diverticula perforation	Hartmann procedure	10 days
Hogendorf [38]	2018	76	F (Case 1)	Unknown	Plastic	CBD stone	6 months	Diverticulosis	Abdominal pain	Sigmoid diverticula perforation	Sigmoidectomy	24 days
		68	M (Case 2)	7 Fr 10 cm straight	Plastic	Pancreas head cancer with CBD invasion	1 month	N/A	Abdominal pain	Rectum	Transverse colostomy	11 days
Riccardi [39]	2019	79	F	10 Fr 10 cm straight, Double pigtail 7 Fr	Plastic	CBD stone	1 month	Diverticulosis	Abdominal pain	Sigmoid diverticula perforation	Hartmann procedure	9 days
Marcos [40]	2020	65	F	10 Fr 5 cm straight	Plastic	Post cholecystectomy bile duct stricture	12 months	Diverticulosis	Asymptomatic	Sigmoid diverticula perforation	Surgical primary closure	Unknown
Pengermä [41]	2021	66	F	10 Fr 5 cm straight	Plastic	Chronic pancreatitis with distal biliary stricture	2 months	N/A	Abdominal pain	Appendix	Appendicectomy	Unknown
Tao [42]	2021	54	M	Straight	Plastic	Acute Cholangitis with CBD stone, biliary pancreatitis	3 months	N/A	Abdominal pain	Sigmoid	Sigmoidectomy + colostomy	10 days
Park [5]	2021	74	M	10 Fr 7 cm straight	Plastic	Acute Cholangitis with CBD stone	1 month	Diverticulosis	Abdominal pain	Ascending colon	Surgical hemicolectomy	Unknown
Ong [43]	2021	57	M	Straight	Plastic	CBD stone	8 years	N/A	Abdominal pain	Caecum	Surgical primary closure	Unknown
Kodia [1]	2022	60	F	Unknown	Plastic	Gallbladder carcinoma with CBD invasion	2 months	N/A	Abdominal pain	Ascending colon	Hybrid laparoendoscopic	4 days
Yamaguchi [44]	2022	86	F	7 Fr 7 cm straight	Plastic	Acute Cholangitis with CBD stone	1 month	Diverticulosis	Abdominal pain	Sigmoid diverticula perforation	Endoscopic removal and clip closure	14 days

A search of literature was conducted using the terms ‘biliary stent’, ‘bowel perforation’, ‘migrated biliary stent’, ‘endoscopic retrograde cholangiopancreatography’ and ‘complications’ across multiple databases including PubMed, SCOPUS and Google Scholar. The inclusion criteria specifically focused on articles that reported colonic perforation as a complication attributed to migratory biliary stents. Articles reporting complications other than colonic perforation were excluded. Of the 101 articles found, 35 articles satisfied the criteria with 37 cases of colonic perforation attributed to migratory biliary stents (Table 1).

The most common site of perforation due to a distally migrated stent was the sigmoid colon (28 out of 37 cases). Diverticular disease was reported in 62% of patients with colon perforation (23 cases). The mean age was 72 years (+/−10.1) with a 9-month (+/−17.7) average time of migration. Of the 36 cases reporting sex, 66% were female (24 out of 36). Out of the 37 cases, the vast majority (33 cases) utilized plastic stents. Plastic stents have a reported higher migration risk when compared with metal [15]. To the best of our knowledge, this is the first reported case of multiple biliary stent migration with two stents of differing material (plastic and metal). Placement of multiple biliary stents decreases the frequency of migration; however, the limited evidence is available in the frequency of migration with using different material stents together [3]. Abdominal pain was the most common clinical presentation reported in 34 of the 37 cases (Table 1). Other complaints included groin swelling, leg pain, pneumaturia and only two patients reported no symptoms at the time of confirmational imaging. Diagnostic CT was utilized in 78% of patients (28 out of 36 cases) to confirm perforation of the gastro-intestinal tract.

Of the 37 reported cases, surgical intervention was used in 27 and eight used endoscopic retrieval with or without clip closure. There was one case describing removal of the biliary stent through a colocutaneous fistula and one utilizing a hybrid laparoendoscopic method. Post-operative complications included abscess formation, post-operative ileus, peritonitis and non-ST elevation myocardial infraction (Table 2). The average length of stay in hospital was 9.6 days (+/−5.6). There was no statistically significant difference for length of stay between surgical and endoscopic intervention (11.2 vs. 6.6, *P* = 0.12). The morbidity rate was ~16.7% (6 out of 36 cases). Of the 34 cases that reported mortality, there was only one fatal outcome attributed to multiorgan failure 5 days post Hartmann’s procedure [12]. There were limitations with the review regarding unknown medical and surgical history of patients along with biochemical results on presentation. Of the 37 cases, approximately only one-quarter stated this information in differing degree in the case report.

**Table 2:** Cases with reported complications post intervention

Author	Intervention	Complication
Alcaide [29]	Endoscopic removal and clip closure	Abscess formation
Chittleborough [35]	Hartmann procedure	Post-operative ileus
Hogendorf [38] (Case 1)	Sigmoidectomy	Peritonitis
Hogendorf [38] (Case 2)	Transverse colostomy	Peritonitis
Riccardi [39]	Hartmann procedure	Non-STEMI
Pengermä [41]	Appendicectomy	Abscess formation

In patients presenting for ERCP, we advocate that risks concerning migratory stents are raised in the consent with possible endoscopic, surgical or non-operative measures. The vast majority of these patients are elderly with high comorbidities. In patients with advanced age and significant comorbidities, endoscopic retrieval of migrated biliary stents may avoid associated morbidity of laparoscopic and laparotomy surgical interventions in cases of perforation without peritonitis. In our case, the rate of post-operative complications for ASA IV is ~9.6 in 1000 and increases to 26.5 in 1000 in emergency surgery cases [14]. Given this the increased risk of surgical intervention, he was not deemed a suitable candidate. Minimally invasive interventions in selected elderly patients as an alternative may facilitate decreased length of stay in hospital and decrease increased morbidity associated with surgical interventions. Clinical judgment regarding a patients’ age, comorbidities and prognosis must be taken into account in deciding whether to opt for surgical intervention or consider alternatives such as endoscopic retrieval or conservative management. Further evidence is needed to assess the frequency of migration in cases of two or more biliary stents that differ in material.

## CONCLUSION

Patients presenting with abdominal pain on a background history of previous ERCP and biliary stenting should raise the suspicion of stent migration. Endoscopic retrieval with or without clip closure may be indicated in hemodynamically stable patients without evidence of peritonitis, abscess and fistula formation. As endoscopic interventions become more utilized in cases of migrated biliary stents, comparison of complications rates can be made against surgical techniques.
